# Does Melissa officinalis consumption improve the salivary antioxidant status of smokers?

**DOI:** 10.22088/cjim.11.4.437

**Published:** 2020

**Authors:** Sedigheh Bakhtiari, Zahra Yadegari, Puriya ehyaei, Narges Gholizadeh

**Affiliations:** 1 Oral and Maxillofacial Medicine, School of Dentistry, Shahid Beheshti University of Medical Sciences, Tehran, Iran; 2 Dental School, Shahid Beheshti University of Medical Sciences, Tehran, Iran; 3 Oral and Maxillofacial Medicine, School of Dentistry, Tehran University of Medical Sciences, Tehran, Iran

**Keywords:** Melissa officinalis, Antioxidant, Saliva, Smoking

## Abstract

**Background:**

The aim of this study was to investigate the effects of *Melissa officinalis* tea on the total antioxidant capacity of saliva among smokers.

**Methods:**

24 smokers were selected by convenience sampling. Demographic information and duration of smoking were recorded at the beginning of study. Two cups of *Melissa officinalis* tea were given to the participants with specific instruction for 30 days. The unstimulated saliva was collected on first day, 15^th^ and 30^th^ days. Then, total antioxidant capacity was measured by a special kit. Statistical analysis was conducted by repeated measure ANOVA test.

**Results:**

The mean values of total antioxidant capacity of saliva were significantly higher in days 15 and 30 from the baseline. (p<0.0001, P=0.006). In day 30, the mean value of antioxidant was not significantly different from day 15. (P=0.271).

**Conclusion:**

*Melissa officinalis* tea consumption increases salivary antioxidants level in smokers.

Cigarette smoke contains a various component such as nicotine, phenol, acetaldehyde, nitric oxide and cadmium. They are assumed as the oxidant and peroxidation materials. Studies showed that smoking reduces the salivary antioxidants and increases the saliva’s oxidative conditions. Saliva is the first biological liquid that is exposed to the smoke of cigarette (1). Since saliva is rich in antioxidants, it can be the first defensive line against oxidative stress. Antioxidant system of salivary consists of enzymatic antioxidant systems such as superoxide dismutase and non-enzymatic as well as uric acid, glutathione. Antioxidants can stop the oxidation process by neutralizing free radicals, to do this, the antioxidants may be oxidized themselves.(2). *Melissa officinalis* is of lamiaceae family and possesses the therapeutic features of meditating, anti-tympanite, anti-spasm, antibacterial, antivirus, anti-inflammatory, antioxidant and neural protection. Essential oil of this planet includes more antioxidants especially the phenol groups. The most common phenol components in this plant include acid rosemary, luteolin 7-o-glucoside, quercetin 3-rutinoside, galic acid and ferulic acid. These components decrease free radicals leading to the reduction in LPO (lipid peroxidation) and decrease or stop MPO (Myelo peroxidation). It finally decreases DNA degeneration in cells and other tissues, additionally, phenol compounds in the *Melissa officinalis* can increase the antioxidant enzymes and their activity (3). Although many studies have been conducted on the antioxidant feature of this herbal nutrient (3.^4^), their results are not comparable with each other because they utilize various methods. This study analyzed the effect of *Melissa officinalis* on TAOC of saliva among smokers.

## Methods

Thirty current smokers (with at least 100 cigarette use in their life and smoked 30 days ago who referred to Oral Medicine Department of Shahid Beheshti University of Medical Sciences (Tehran, Iran)) were included in the study. This study was approved by Ethics Committee of Shahid Beheshti University of Medical Sciences (ethical code number IR.SBMU.RIDS.REC.1395.271( Demographic information and duration of smoking were recorded in the questionnaire.

 Exclusion criteria were as follows: systemic disease, pregnancy, history of chemotherapy or radiotherapy, take any routine medications during past 3 months, consuming alcohol and any other types of smoking or tobacco products, changes in diet or daily activity at any time after study, consumption of antioxidant supplements such as vitamins E and C, consumption of other sources of antioxidant such as green tea components. Participants were asked to consume two cups of pure herbal tea (*Melissa officinalis* tea bags, Darband Tea Company, Iran) per day for 30 days. Each tea bag contained 2 gr. pure *Melissa officinalis.* The herbal tea was put in 150 ml hot water (around 80 ºC) for 5 min before consumption (5). Each participant was given an equal-sized cup. The participants were asked to drink the herbal tea between breakfast and lunch and between lunch and dinner, and avoid drinking it with empty stomach. Adding sugar was no exception but milk is prohibited. They were asked also to avoid changing their diet during the study. Participants who could not comply this protocol have been excluded from the study. Therefore, we reached to 24 participants by the end of the study.

The saliva samples were collected on the first day, 15^th^ and 30^th^ day of study before the first meal of consuming herbal tea on that day. The participants should avoid eating, drinking and smoking for two hours before saliva collection. By spitting method, unstimulated saliva samples were collected between 9 A.M. and 12 A.M. After washing their mouth with 15 ml distilled water. The samples were inserted into the 15 ml falcon tubes and were centrifuged for 15 minutes at 3000 × g. Then the clarified supernatant was collected in microtubes with the specified codes and they were frozen at – 70 ºC until the day of assessment. After thawing the samples at room temperature and to resolve any cryoprecipitate, they were mixed by a vortex mixer for 20 seconds gently. TAOC were analyzed with the zellBio GmbH (CAT No: zb-tac-96a, Germany) kit (mmol/l) using ELISA reader (Anthos 2020, Austria) in the wavelength of 490 nm. To increase the validity of study, all samples were assayed in duplicate based on the kit’s instruction. The amount of TAOC of each sample was calculated according to the standard curve. The values for mean and standard deviation are achieved by means of repeated measure ANOVA and Bonferroni tests to compare the TAOC of saliva in different times.

## Results

Thirty smoker cases between ages 25 to 40 years participated in this study but 6 people were excluded. The final participants included 19 men (age range 25-40) and 5 women (age range 30-38). On average, they smoked 7 cigarettes every day. None of participants used any other smoking or tobacco products. Table 1 shows the type of cigarette, the frequency, and nicotine value as well as numbers in each packet (based on information on packets).

**Table 1:** Frequency of consuming cigarette, nicotine extent and number of cigarettes

**Tar** **(mg)**	**Nicotine Extent (mg)**	**Number of cigarettes**	**Type of cigarette**
12	0.7	12	Winston
4.5	0.45	4	Esse
6	0.5	4	Marlboro
8	0.7	2	Bahman
14	0.8	1	Magna
5	0.6	1	Montana

The salivary TAOC was measured 0.163 ±.098 at the beginning of the study. After 15 days of consuming *Melissa officinalis* tea, the amount of TAOC means reached 0.229±0.13. On day 30^th^, TAOC mean was 0.205 ±0.10. Kolmogorov–Smirnov test and Shapiro–Wilk test showed that the distribution of TAOC values in groups follows the normal distribution (p>0.05). In repeated measure ANOVA analysis, the sphericity assumption was met by the data (P=0.607). The results of repeated measure ANOVA showed that the mean of TAOC values were different between at least 2 time points. By performing pairwise comparison and applying Bonferroni correction, we found that the mean TAOC values were significantly higher in day 15^th^ and 30^th^ compared with the baseline (p<0.0001, P=0.006). The mean value of TAOC was a bit lower in day 30^th ^compared with day 15^th^, but it was not significant (P=0.271) (table 2).

**Table 2:** Comparing the mean of TAOC of saliva on different days

		**Mean Difference**	**Std. Error**	**P-value**
T1	T2	-0.066^*^	0.015	<0.0001
T1	T3	-0.042^*^	0.012	0.006
T2	T3	0.024	0.014	0.271

T1= first day, T2= 15^th^ day, T3= 30^th ^day

The significance level has been considered at a p≤ 0.05.

## Discussion

In this study, we evaluated the effect of consuming *Melissa Officinalis* tea 4gr a day (2 bags * 2 gr) on TAOC value among the smokers’ saliva .and our results showed that consuming Melissa officinalis tea increased the TAOC of saliva. The antioxidants protect the body against the reactive oxygen species (ROS) radicals. Nowadays focus has been shifted to the consumption of antioxidant nutrition such as flavonoids, phenolic acids, and tocopherols because these materials are cheaper, more accessible and have higher antioxidant feature and show lower toxicity in contrast to the synthetic materials (6). A few studies have been conducted regarding salivary oxidative and antioxidant activities. They have shown that saliva can be very useful in diagnosing the effective conditions on the oxidative-antioxidant system (7). 

Zeraatpisheh et al. 2011 evaluated the effect of *Melissa officinalis* on oxidative stress resulting from low and continuous dosages of x- ray on employees of radiology ward after 15 days of consuming *Melissa officinalis*. They observed that consuming *Melissa officinalis* supplements improved significantly the condition of oxidative stresses and DNA degeneration among clerks of radiology after 30 days (8). According to our results. Between 15 and 30 days of use, the TAOC decreased, but this decrease was not significant and it seemed to be due to the total salivary antioxidant capacity reaching to maximum on day 15 and the creation of Plateau state in the curve of the antioxidant capacity. In general, 30 days after the use of *Melissa officinalis*, as in the case study, we observed an improvement in the antioxidant capacity.

Bakhtiari et al. (2012) investigated the effect of vitamin C on TAOC of saliva among smokers and reported that vitamin C cannot significantly affect the TAOC of saliva after a 3-week consumption, but TAOC increased in the two first weeks (9). Azimi et al. (2017) analyzed the effect of green tea on TAOC of saliva among smokers and reported that green tea improves effectively the antioxidant condition of saliva among smokers (10). Similar to Azimi et al.’s study, we analyzed the TAOC of saliva because the single measurement of antioxidants was very time-consuming and some of them may be unknown yet. Additionally, their reciprocal effects were overlooked.

Several studies evaluated the antioxidant characteristics of *Melissa officinalis*. One study analyzed the antioxidant characteristic of 6 members of laminaceae including dittany, mint, sage, siderites, sweet majoram, and lemon balm. The aqueous extract of Melissa officinalis contains much value of phenol compounds and significantly affects the prevention of lipids’ oxidation (11). Kamdem et al. (2013) investigated the antioxidant characteristic of Melissa officinalis and found a direct relationship between TAOC and *Melissa officinalis* density. They observed that its ethanol extract does not have cytotoxicity effect on human WBCs (12). Totally, our findings are in agreement with the results of previous studies, investigating antioxidant effect of *Melissa officinalis.*

In conclusion the results of this study indicated that TAOC of saliva among smokers increased significantly under the effect of consuming *Melissa officinalis*. Further clinical trial studies are required to evaluate the therapeutic value of antioxidants in smoker patients.
